# Ensemble-SINDy: Robust sparse model discovery in the low-data, high-noise limit, with active learning and control

**DOI:** 10.1098/rspa.2021.0904

**Published:** 2022-04

**Authors:** U. Fasel, J. N. Kutz, B. W. Brunton, S. L. Brunton

**Affiliations:** ^1^ Department of Mechanical Engineering, University of Washington, Seattle, WA, USA; ^2^ Department of Applied Mathematics, University of Washington, Seattle, WA, USA; ^3^ Department of Biology, University of Washington, Seattle, WA, USA

**Keywords:** nonlinear dynamics, sparse regression, model discovery, ensemble methods, probabilistic forecasting, active learning

## Abstract

Sparse model identification enables the discovery of nonlinear dynamical systems purely from data; however, this approach is sensitive to noise, especially in the low-data limit. In this work, we leverage the statistical approach of bootstrap aggregating (bagging) to robustify the sparse identification of the nonlinear dynamics (SINDy) algorithm. First, an ensemble of SINDy models is identified from subsets of limited and noisy data. The aggregate model statistics are then used to produce inclusion probabilities of the candidate functions, which enables uncertainty quantification and probabilistic forecasts. We apply this ensemble-SINDy (E-SINDy) algorithm to several synthetic and real-world datasets and demonstrate substantial improvements to the accuracy and robustness of model discovery from extremely noisy and limited data. For example, E-SINDy uncovers partial differential equations models from data with more than twice as much measurement noise as has been previously reported. Similarly, E-SINDy learns the Lotka Volterra dynamics from remarkably limited data of yearly lynx and hare pelts collected from 1900 to 1920. E-SINDy is computationally efficient, with similar scaling as standard SINDy. Finally, we show that ensemble statistics from E-SINDy can be exploited for active learning and improved model predictive control.

## 1. Introduction

Data-driven model discovery enables the characterization of complex systems where first principles derivations remain elusive, such as in neuroscience, power grids, epidemiology, finance and ecology. A wide range of data-driven model discovery methods exist, including equation-free modelling [1], normal form identification [2–4], nonlinear Laplacian spectral analysis [5], Koopman analysis [6,7] and dynamic mode decomposition (DMD) [8–10], symbolic regression [11–15], sparse regression [16,17], Gaussian processes [18], combining machine learning and data assimilation [19,20], and deep learning [21–27]. Limited data and noisy measurements are fundamental challenges for all of these model discovery methods, often limiting the effectiveness of such techniques across diverse application areas. The *sparse identification of nonlinear dynamics* (SINDy) [16] algorithm is promising, because it enables the discovery of interpretable and generalizable models that balance accuracy and efficiency. Moreover, SINDy is based on simple sparse linear regression that is highly extensible and requires significantly less data in comparison with, for instance, neural networks. In this work, we unify and extend innovations of the SINDy algorithm by leveraging classical statistical bagging methods [28] to produce a computationally efficient and robust probabilistic model discovery method that overcomes the two canonical failure points of model discovery: noise and limited data.

The SINDy algorithm [16] provides a data-driven model discovery framework, relying on sparsity-promoting optimization to identify parsimonious models that avoid overfitting. These models may be ordinary differential equations (ODEs) [16] or partial differential equations (PDEs) [17,29]. SINDy has been applied to a number of challenging model discovery problems, including for reduced-order models of fluid dynamics [30–35] and plasma dynamics [36–38], turbulence closures [39–41], mesoscale ocean closures [42], nonlinear optics [43], computational chemistry [44] and numerical integration schemes [45]. SINDy has been widely adopted, in part, because it is highly extensible. Extensions of the SINDy algorithm include accounting for control inputs [46] and rational functions [47,48], enforcing known conservation laws and symmetries [30], promoting stability [49], improved noise robustness through the integral formulation [37,50–54], generalizations for stochastic dynamics [44,55] and tensor formulations [56], and probabilistic model discovery via sparse Bayesian inference [57–61]. Many of these innovations have been incorporated into the open source software package PySINDy [62,63]. Today, the biggest challenge with SINDy, and more broadly in model discovery, is learning models from limited and noisy data, especially for spatio-temporal systems governed by PDEs.

Model discovery algorithms are sensitive to noise because they rely on the accurate computation of derivatives, which is especially challenging for PDEs where noise can be strongly amplified when computing higher-order spatial derivatives. There have been two key innovations to improve the noise robustness of SINDy: control volume formulations and ensemble methods. The integral formulation of SINDy [50] has proven powerful, enabling the identification of PDEs in a weak form that averages over control volumes, which significantly improves its noise tolerance. This approach has been used to discover a hierarchy of PDE models for fluids and plasmas [37,51–54,64,65]. Several works have begun to explore ensemble methods to robustify data-driven modelling, including the use of bagging for DMD [66], ensemble-Lasso [67], subsample aggregating for improved discovery [61,68], statistical learning of PDEs to select model coefficients with high importance measures [69] and improved discovery using ensembles based on subsampling of the data [51,52,61,65]. Also, symbolic regression methods [11–13] and spectral proper orthogonal decomposition (SPOD) [70] are inherently imbued with ensembling ideas. Symbolic regression models are formed by initially randomly combining mathematical building blocks (library terms) and then recombining building blocks (equations or library terms) that best model the experimental data. In SPOD, a modal decomposition method closely related to DMD, optimally averaged DMD modes are obtained from an ensemble DMD problem. Thus, both these methods naturally include ensembling ideas.

When dealing with noise-compromised data, it is also critical to provide uncertainty estimates of the discovered models. In this direction, recent innovations of SINDy use sparse Bayesian inference for probabilistic model discovery [57–60]. Such methods employ Markov Chain Monte Carlo, which is extremely computationally intensive. These extensions have all improved the robustness of SINDy for high-noise data, although they have been developed largely in isolation and they have not been fully characterized, exploited and/or integrated.

In this work, we unify and extend recent innovations in ensembling and the weak formulation of SINDy to develop and characterize a more robust ensemble-SINDy (E-SINDy) algorithm. Furthermore, we show how this method can be useful for active learning and control. In particular, we apply b(r)agging^1^ to SINDy to identify models of nonlinear ODEs of the form

1.1 $$\frac{\text{d}}{\text{dt}}\text{u}=\text{f}(\text{u}),\;\text{u}(0)={\text{u}}_{0},$$

with state $u\in R^{n}$ and dynamics f(u), and for nonlinear PDEs of the form

**Figure 1.**
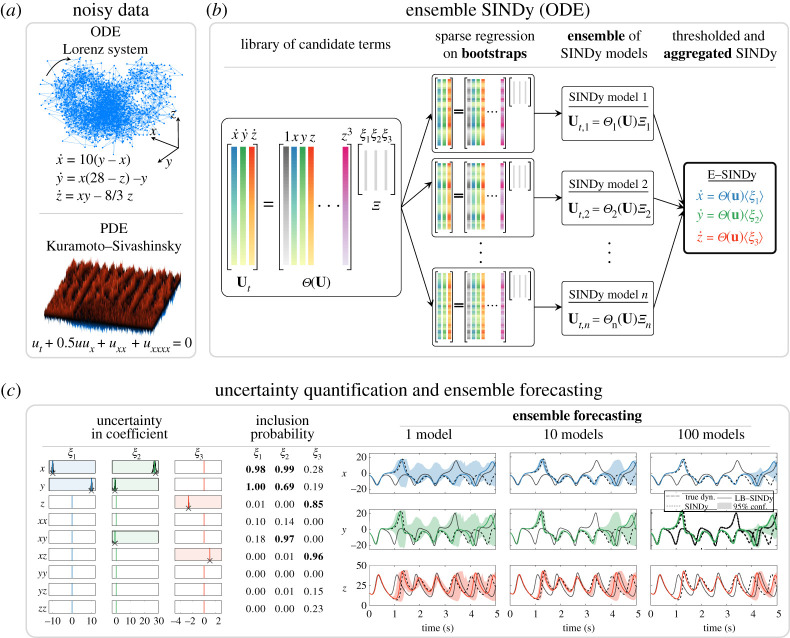
(*a*–*c*) Schematic of the E-SINDy framework. E-SINDy exploits the statistical method of bootstrap aggregating (bagging) to identify ordinary and partial differential equations that govern the dynamics of observed noisy data. First, sparse regression is performed on bootstraps of the measured data, or on the library terms in case of library bagging, to identify an ensemble of SINDy models. The mean or median of the coefficients are then computed, coefficients with low inclusion probabilities are thresholded, and the E-SINDy model is aggregated and used for forecasting. (Online version in colour.)

1.2 $${\text{u}}_{t}=\text{N}(\text{u},{\text{u}}_{x},{\text{u}}_{xx},\ldots ,x,\mu ),$$

with N(⋅) a system of nonlinear functions of the state u(x,t), its derivatives and parameters μ; partial derivatives are denoted with subscripts, such that $u_{t}:=\partial u/\partial t$. We show that b(r)agging improves the accuracy and robustness of SINDy. The method also promotes interpretability through the inclusion probabilities of candidate functions, enabling uncertainty quantification. Importantly, the ensemble statistics are useful for producing probabilistic forecasts and can be used for active learning and nonlinear control. We also demonstrate library E-SINDy, which subsamples terms in the SINDy library. E-SINDy is computationally efficient compared with probabilistic model identification methods based on Markov Chain Monte Carlo sampling [60], which take several hours of CPU time to identify a model. By contrast, our method identifies models and summary statistics in seconds by leveraging the sparse regression of SINDy with statistical bagging techniques. Indeed, E-SINDy has similar computational scaling to standard SINDy. This method applies under the same conditions as the standard SINDy algorithm, where it is assumed that all relevant variables are measured at a sufficient temporal and spatial resolution so as to approximate derivatives. We investigate different ensemble methods, apply them to several synthetic and real-world datasets, and demonstrate that E-SINDy outperforms existing sparse regression methods, especially in the low-data and high-noise limit. A schematic of E-SINDy is shown in figure 1. We first describe SINDy for ODEs and PDEs in §2, before introducing the E-SINDy extension in §3, and discussing applications to challenging model discovery, active learning and control problems in §4.

## 2. Background

Here, we describe SINDy [16], a data-driven model discovery method to identify sparse nonlinear models from measurement data. First, we introduce SINDy to identify ODEs, followed by its generalization to identify PDEs [17,29].

### (a) Sparse identification of nonlinear dynamics

The SINDy algorithm [16] identifies nonlinear dynamical systems from data, based on the assumption that many systems have relatively few active terms in the dynamics f in equation (1.1). SINDy uses sparse regression to identify these few active terms out of a library of candidate linear and nonlinear model terms. Therefore, sparsity-promoting techniques may be used to find parsimonious models that automatically balance model complexity with accuracy [16]. We first measure m snapshots of the state u in time and arrange these into a data matrix

2.1 $$\text{U}={\left[{\text{u}}_{1}\;{\text{u}}_{2}\;\cdots \;{\text{u}}_{m}\right]}^{T}.$$

Next, we compute the library of D candidate nonlinear functions $\Theta (U)\in R^{m\times D}$

2.2 $$\Theta (\text{U})=[\text{1}\;\text{U}\;{\text{U}}^{2}\;\cdots \;{\text{U}}^{d}\;\cdots \;\sin(\text{U})\;\cdots ].$$

This library is constructed to include any functions that might describe the data, and this choice is crucial. The underlying dynamical system is unknown and we cannot guarantee that the dynamics are well described by the span of the library. Therefore, the recommended strategy is to start with a basic choice, such as low-order polynomials, and then increase the complexity and order of the library until sparse and accurate models are obtained.

We must also compute the time derivatives of the state $U_{t}={\left[{\dot{u}}_{1}\;{\dot{u}}_{2}\;\cdots \;{\dot{u}}_{m}\right]}^{T}$, typically by numerical differentiation. We therefore need a suitable data sampling time that allows for the computation of the time derivatives, which may limit the applicability of the SINDy algorithm for certain datasets with coarse or uneven sampling in time. The system in equation (1.1) may then be written in terms of these data matrices

2.3 $${\text{U}}_{t}=\Theta (\text{U})\Xi .$$

Each entry in $\Xi \in R^{D\times n}$ is a coefficient corresponding to a term in the dynamical system. Many dynamical systems have relatively few active terms in the governing equations. Thus, we may employ sparse regression to identify a sparse matrix of coefficients Ξ signifying the fewest active terms from the library that result in a good model fit

2.4 $$\Xi ={\text{arg min}}_{\hat{\Xi }}\frac{1}{2}||{\text{U}}_{t}-\Theta (\text{U})\hat{\Xi }|{|}_{2}^{2}+R(\hat{\Xi }).$$

The regularizer R(Ξ) is chosen to promote sparsity in Ξ. For example, sequentially thresholded least-squares (STLS) [16] uses $R(\Xi )=\lambda ||\Xi |{|}_{0}$ with a single hyperparameter λ, whereas sequentially thresholded ridge regression (STRidge) [17] uses $R(\Xi )=\lambda_{1}||\Xi |{|}_{0}+\lambda_{2}||\Xi |{|}_{2}$ with two hyperparameters $\lambda_{1}$ and $\lambda_{2}$. STLS was first introduced to discover ODEs and STRidge was introduced to discover PDEs where data can be highly correlated and STLS tends to perform poorly. There are several other recently proposed regularizers and optimization schemes [49,71,72]. We illustrate STRidge in pseudo code algorithm 1, noting that STRidge reduces to STLS for $\lambda_{2}=0$.


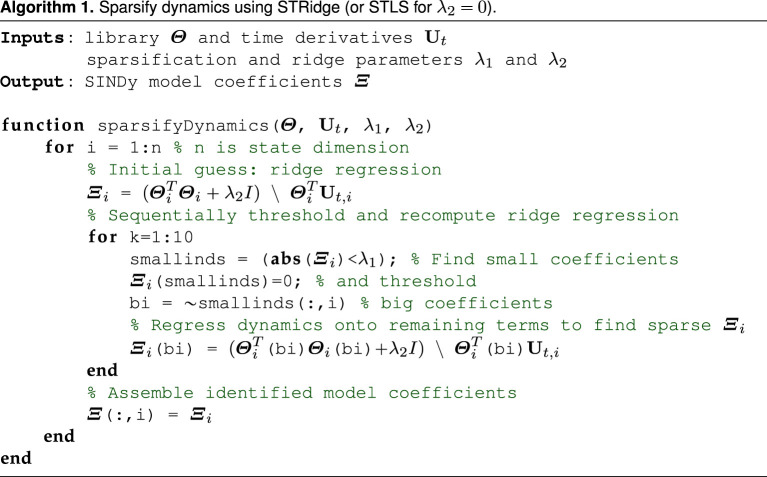


### (b) Discovering PDEs

SINDy was recently generalized to identify PDEs [17,29] in the *partial differential equation functional identification of nonlinear dynamics* (PDE-FIND) algorithm. PDE-FIND is similar to SINDy, but with the library including partial derivatives. Spatial time-series data are arranged into a column vector $U\in R^{mn}$, with data collected over m time points and n spatial locations. Thus, for PDE-FIND, the library of candidate terms is $\Theta (U)\in R^{mn\times D}$. The PDE-FIND implementation of Rudy *et al.* [17] takes derivatives using finite difference for clean data or polynomial interpolation for noisy data. The library of candidate terms can then be evaluated:

2.5 $$\Theta (\text{U})=[\text{1}\;\text{U}\;{\text{U}}^{2}\;\cdots \;{\text{U}}_{x}\;\cdots \;{\text{UU}}_{x}\;\cdots ].$$

The time derivative $U_{t}$ is reshaped into a column vector and the system in equation (1.2) is written as

2.6 $${\text{U}}_{t}=\Theta (\text{U})\Xi .$$

For most PDEs, Ξ is sparse and can be identified with a similar sparsity-promoting regression

2.7 $$\Xi ={\text{arg min}}_{\hat{\Xi }}\frac{1}{2}||{\text{U}}_{t}-\Theta (\text{U})\hat{\Xi }|{|}_{2}^{2}+R(\hat{\Xi }).$$

STRidge improves model identification with highly correlated data that is common in PDE regression problems. PDE-FIND is extremely prone to noise, because noise is amplified when computing high-order partial derivatives for Θ. To make PDE-FIND more noise robust, integral [50] and weak formulations [51,54] were introduced. Instead of discovering a model based on equation (1.2), the PDE can be multiplied by a weight $w_{j}(u,t)$ and integrated over a domain $\Omega_{k}$. This can be repeated for a number of combinations of $w_{j}(u,t)$ and $\Omega_{k}$. Stacking the results of the integration over different domains using different weights leads to a linear system

2.8 $${\text{q}}_{0}=\text{Q}\Xi ,$$

with $q_{0}$ and $Q=[q_{1},\ldots ,q_{D}]$ the integrated left-hand side and integrated library of candidate terms, which replace ${\text{U}}_{t}$ and the library of nonlinear functions Θ(U). As with PDE-FIND, sparse regression can be employed to identify a sparse matrix of coefficients Ξ, using STLS, STRidge or other regularizers. For all of our results, we use this weak formulation as a baseline and for the basis of ensemble models.

## 3. Ensemble SINDy

In this work, we introduce E-SINDy, which incorporates ensembling techniques into data-driven model discovery. Ensembling is a well-established machine learning technique that combines multiple models to improve prediction. A range of ensembling methods exist, such as bagging (bootstrap aggregation) [28], bragging (robust bagging) [73,74] and boosting [75,76]. Structure learning techniques such as cross-validation [77] or stability selection [78] can also be considered ensembling methods, because they combine and use the information of a collection of learners or models. For model discovery, ensembling improves robustness and naturally provides inclusion probabilities and uncertainty estimates for the identified model coefficients, which enable probabilistic forecasting and active learning.

**Figure 2.**
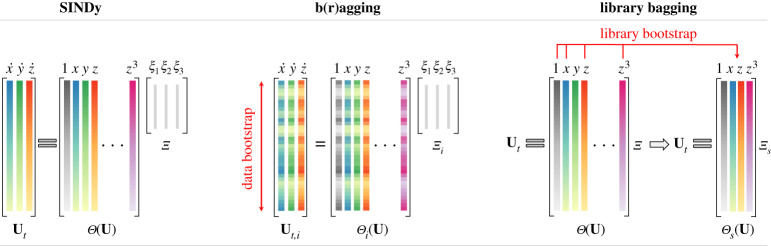
Schematic of SINDy and E-SINDy with b(r)agging and library bagging. Shown is a single model of the ensemble. In the case of b(r)agging, data bootstraps (data samples with replacement) are used to generate an ensemble of SINDy models. The E-SINDy model is aggregated by taking the mean of the identified coefficients for bagging, and the median for bragging. In case of library bagging, instead of data bootstraps, library term bootstraps are sampled without replacement. Library terms with low inclusion probability are discarded and the E-SINDy model can be identified on the smaller library using standard SINDy or b(r)agging E-SINDy. (Online version in colour.)

Here, we propose two new ensemble model discovery methods: the first method is called b(r)agging E-SINDy, and the second method is called library E-SINDy. A general schematic of E-SINDy is shown in figure 1, and a schematic of the sparse regression problems for b(r)agging and library E-SINDy is shown in figure 2. Our first method, b(r)agging E-SINDy, uses data bootstraps to discover an ensemble of models that are aggregated by taking the mean of the identified model coefficients in case of bagging, and taking the median in the case of bragging. Bootstraps are data samples with replacement. Applied to SINDy to identify ODEs, we first build a library of candidate terms Θ(U) and derivatives ${\text{U}}_{t}$. From the m rows of the data matrices ${\text{U}}_{t}$ and Θ(U), corresponding to m samples in time, we select q bootstrapped data samples and generate q SINDy models in the ensemble. For each of these q data bootstraps, m new rows are sampled with replacement from the original m rows of the data matrices. On average, each data bootstrap will have around 63% of the entries of the original data matrices, with some of these entries being represented multiple times in the bootstrap; for large m this quantity converges to $1-e^{-1}\approx 0.632$, which is the limit of $1-{\left(1-(1/m)\right)}^{m}$ for m→∞. In this way, randomness and subsampling is inherent to the bootstrapping procedure. From the q identified SINDy models in the ensemble, we can either directly aggregate the identified models, or first threshold coefficients with low inclusion probability. The procedure is illustrated in algorithm 2 for bagging E-SINDy using STRidge. The same procedure applies for bragging, taking the median instead of the mean, and using other regularizers than STRidge. Note that there are other random data subsampling approaches that may be used, such as generating q models based on q random subsamples of p<m rows of the data without replacement, of which there are (mp). However, boostrapping based on selection with replacement is the most standard procedure.


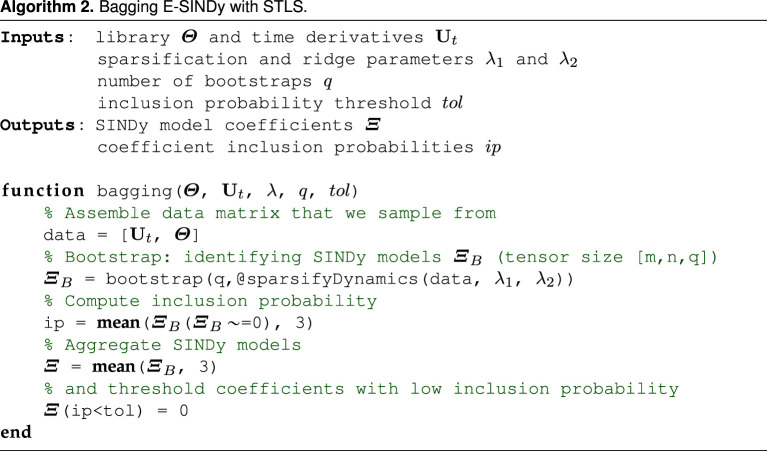


The second method proposed, library bagging E-SINDy, samples library terms instead of data pairs. We sample l out of D library terms without replacement. In case of sampling library terms, replacement does not affect the sparse regression problem. However, using smaller libraries can drastically speed up model identification, as the complexity of the least-squares algorithm is $O(ml^{2})$. Library bagging with small l can therefore help counteract the increased computational cost of solving multiple regression problems in the ensemble. As with bagging E-SINDy, we obtain an ensemble of models and model coefficient inclusion probabilities. We can directly aggregate the models and threshold coefficients with low inclusion probabilities to get a library E-SINDy model. We can also use the inclusion probabilities to threshold the library, only keeping relevant terms, and run bagging E-SINDy using the smaller library. This can be particularly useful if we start with a large library: we first identify and remove all library terms that are clearly not relevant and then run bagging E-SINDy on the smaller library. However, the library bagging inclusion probability threshold needs to be selected carefully to not remove relevant terms from the library. We show a pseudo code of library bagging E-SINDy in algorithm 3.


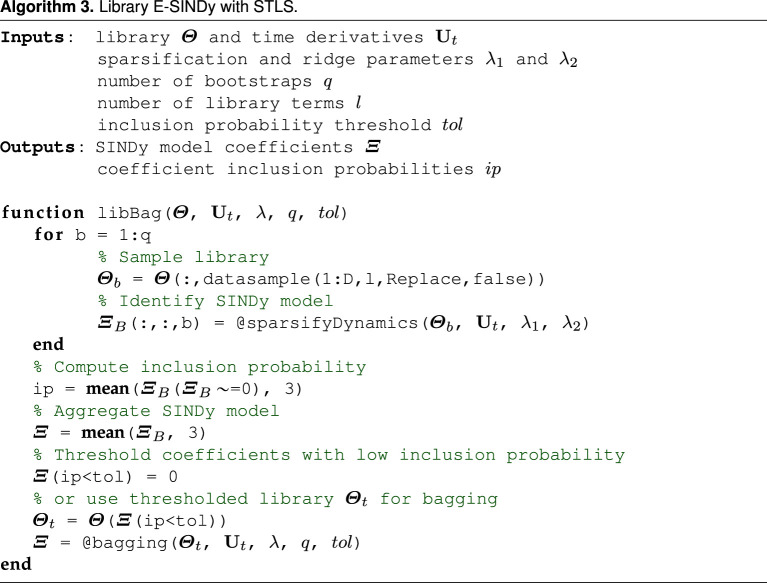


E-SINDy provides inclusion probabilities and uncertainty estimates for the discovered model coefficients, thus connecting to Bayesian model identification techniques. The identified ensemble of model coefficients can be used to compute coefficient probability density functions, which form a posterior distribution p(Ξ|X). In terms of forecasting, we can either use the aggregated mean or median of the identified coefficients to forecast, or we can draw from multiple identified SINDy models to generate ensemble forecasts that represent posterior predictive distributions p(x(t)|X) that provide prediction confidence intervals.

## 4. Results

We now apply E-SINDy to challenging synthetic and real-world datasets to identify ODEs and PDEs. We apply library bagging E-SINDy to a real-world ecological dataset, showing its performance in the very low data limit. For PDEs, we use the recent weak-SINDy (WSINDy) [54] as a baseline and show the improved noise robustness when using E-SINDy for identifying a range of PDEs. Trends for the noise and data length sensitivity of bagging, bragging and library bagging to identify the chaotic Lorenz system dynamics are presented in appendix A.

### (a) ODEs

**Figure 3.**
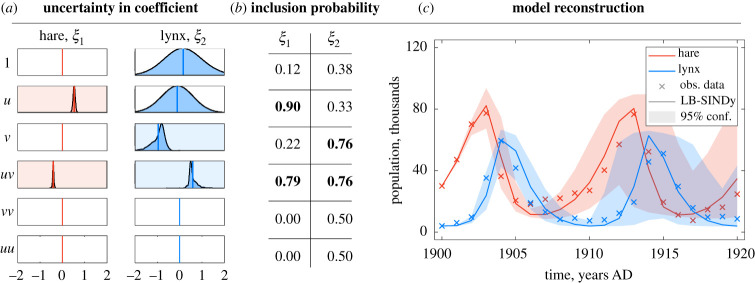
Library bagging E-SINDy (LB-SINDy) on real data: data consisting of measurements by the Hudson Bay Company of lynx and hare pelts from 1900 to 1920. (*a*) Uncertainty in the identified model coefficients, (*b*) inclusion probabilities of the model coefficients (with 65% threshold) and (*c*) model reconstruction. LB-SINDy (continuous lines) uses the mean value of the identified coefficients for reconstruction, and the 95% confidence interval depicts ensemble reconstruction, drawing five models and averaging the coefficients for 1000 realizations. (Online version in colour.)

We apply E-SINDy to a challenging real-world dataset from the Hudson Bay Company, which consists of the yearly number of lynx and hare pelts collected from 1900 to 1920. These pelt counts are thought to be roughly proportional to the population of the two species [79]. Lynx are predators whose diet depends on hares. The population dynamics of the two species should, therefore, be well approximated by a Lotka–Volterra model. There are several challenges in identifying a SINDy model from this dataset: there are only 21 data points available, and there is large uncertainty in the measurements arising from weather variability, consistency in trapping and other changing factors over the years measured. In figure 3, we show that E-SINDy correctly identifies the Lotka–Volterra dynamics, providing model coefficient and inclusion probabilities and confidence intervals for the reconstructed dynamics. We use library bagging, followed by bagging using a library of polynomials up to third order, to identify a sparse model in this very low data limit with only 21 data points per species. Similar results for the lynx-hare dataset were recently published using a probabilistic model discovery method [60] based on sparse Bayesian inference. This approach employed Markov Chain Monte Carlo, for which the computational effort to generate a probabilistic model is comparably high, taking several hours of CPU time. By contrast, E-SINDy takes only seconds to identify a model and its coefficient and inclusion probabilities.

### (b) PDEs

In this section, we present results applying E-SINDy to discover PDEs from noisy data. We use the recent WSINDy implementation [54] as the baseline model for ensembling. WSINDy was successfully applied to identify models in the high-noise regime using large libraries. We perform library bagging on the system of equation (2.8) instead of equation (2.6), and refer to the resulting method as ensemble weak SINDy (E-WSINDy).

We apply E-WSINDy to identify PDEs from synthetic data for the inviscid Burgers, Korteweg de Vries, nonlinear Schroedinger, Kuramoto–Sivashinsky and reaction–diffusion equations. Details on the numerical methods for creating the data are discussed in appendix B and in our E-SINDy data and code repository. We quantify the accuracy and robustness of the model identification by assessing the success rate and model coefficient errors for a number of noise realizations. The success rate is defined as the rate of identifying the correct non-zero and zero terms in the library, averaged over all realizations. The model coefficient error $E_{c}$ quantifies how much the identified coefficients $\hat{\Xi }$ deviate from the true parameters Ξ that we use to generate the data:

4.1 $$E_{c}=\frac{||\Xi -\hat{\Xi }|{|}_{2}}{||\Xi |{|}_{2}}.$$

**Figure 4.**
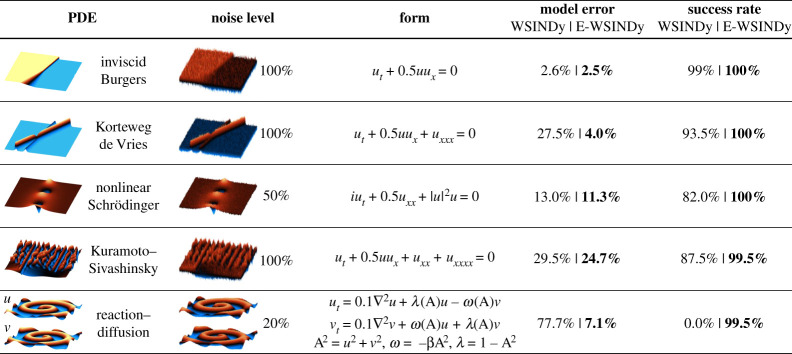
Comparison of model error and success rate of discovered PDEs using weak-SINDy and ensemble weak-SINDy. Ensembling robustifies and improves the accuracy of PDE identification. (Online version in colour.)

The results are summarized in figure 4. For all PDEs, E-WSINDy reduces the model coefficient error and increases the success rate of the model discovery. Moreover, E-WSINDy can accurately identify the correct model structure for the reaction–diffusion case, where WSINDy falsely identifies a linear oscillator model instead of the nonlinear reaction–diffusion model. To investigate the limits of E-WSINDy, we further increase the noise level for each case up to the point where the success rate drops below 90%. On average, for all investigated PDEs, we find that ensembling improves the noise robustness of WSINDy by a factor of 2.3. We conclude that ensembling significantly improves model discovery robustness and enables the identification of PDEs in the extreme noise limit.

### (c) Exploiting ensemble statistics for active learning

We now present results exploiting the ensemble statistics for active learning [80,81]. Active learning is a machine learning method that can reduce training data size while maintaining accuracy by actively exploring regions of the feature space that maximally inform the learning process [82,83]. This can be particularly effective for systems with large feature spaces that are expensive to explore, such as in biological systems or high-dimensional systems with control. In biological systems, collecting samples can be time consuming and expensive, but it may be possible to initialize specific new initial conditions of the system. Active learning can inform the selection of these initial conditions for improved data efficiency of the learning process and model discovery. For control problems, similarly, exploration of large feature spaces may be expensive, such as repeatedly performing robotics experiments. Active learning can enable data-efficient exploration by the guided collection of relevant and descriptive data that optimally supports the model discovery process of the controlled robotic system. Here, we leverage the ensemble statistics of E-SINDy to identify and sample high-uncertainty regions of phase space that maximally inform the sparse regression. In E-SINDy, we collect data from a single initial condition or from multiple randomly selected initial conditions and identify a model in one shot. Instead, we can successively identify E-SINDy models and exploit their ensemble statistics to identify new initial conditions with high information content, which can improve the data efficiency of the model discovery process. The basic idea is to compute ensemble forecasts from a large set of initial conditions using successively improved E-SINDy models and only explore regions with high ensemble forecast variance. Our simple but effective active E-SINDy approach iteratively identifies models in three steps: (1) collecting a small amount of randomly selected data to identify an initial E-SINDy model; (2) selecting a number of random initial conditions and computing the ensemble forecast variance for each initial condition using the current E-SINDy model; and (3) sampling the true system with the initial condition with highest variance. Finally, we concatenate the newly explored data to the existing dataset to identify a new E-SINDy model, and continue the model identification until the model accuracy and/or variance of the identified model coefficients converge.

**Figure 5.**
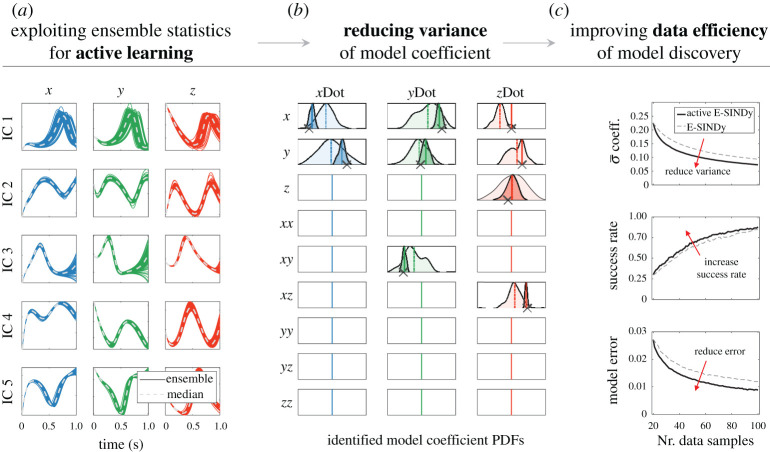
Exploiting ensemble statistics for active learning. Active E-SINDy randomly selects a number of initial conditions (IC), computes the ensemble forecast variance at each IC and explores the IC with highest variance. (*a*) Ensemble forecasts from different ICs. (*b*) Reduced variance of identified model coefficients after several active learning steps. (*c*) Improved data efficiency and accuracy of model discovery using active learning. (Online version in colour.)

Here, we test active E-SINDy on the Lorenz system dynamics introduced in figure 1 and appendix A and show the results in figure 5. In figure 5*a*, we show five illustrative ensemble forecasts from different initial conditions after initializing the algorithm. In total, at each iteration, we compute ensemble forecasts at 200 different initial conditions. We found that at each initial condition, integrating a single time step forward in time is informative enough to compute ensemble forecasting variance. In figure 5*b*, we show the probability density functions of the identified model coefficients at initialization have wide distributions, and after 80 active learning steps the variance of the distributions is significantly reduced. Figure 5*c* also shows the improved data efficiency of the model discovery using active learning E-SINDy compared with E-SINDy. Through active E-SINDy, we reduce the variance of the identified model coefficients, increase the success rate of identifying the correct model structure and reduce the model coefficient error compared with standard E-SINDy.

### (d) Ensemble sparse identification of nonlinear dynamics model predictive control

It is also possible to use E-SINDy to improve model predictive control (MPC) [84–86]. MPC is a particularly compelling approach that enables control of strongly nonlinear systems with constraints, multiple operating conditions and time delays. The major challenge of MPC lies in the development of a suitable model. Deep neural network models have been increasingly used for deep MPC [87,88]; however, they often rely on access to massive datasets, have limited ability to generalize, do not readily incorporate known physical constraints and are computationally expensive. Kaiser *et al.* [46] showed that sparse models obtained via SINDy perform nearly as well with MPC, and may be trained with relatively limited data compared to a neural network. Here, we show that E-SINDy can further reduce the training data requirements compared to SINDy, enabling the control of nonlinear systems in the very low data limit.

**Figure 6.**
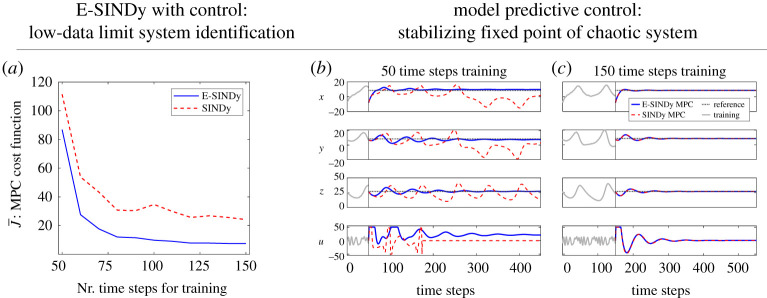
System identification in the low-data limit for model predictive control (MPC). (*a*) MPC cost function average $\bar{J}$ over number of time steps used for training with E-SINDy (blue continuous line) and SINDy (red dashed line). Controlled trajectory coordinates *x*, *y*, z of Lorenz system with MPC input *u*, for models trained with E-SINDy (blue continuous line) and SINDy (red dashed line) using (*b*) 50 and (*c*) 150 time-step data points. (Online version in colour.)

We use E-SINDy to identify a model of the forced Lorenz system dynamics and use MPC to stabilize one of the two unstable fixed points $\left(\pm \sqrt{72},\pm \sqrt{72},27\right)$. The Lorenz system is introduced in figure 1 and we add a control input u to the first state of the dynamics: $\dot{x}=\sigma (y-x)+u$. The control problem is based on Kaiser *et al.* [46]. We describe the MPC problem in more detail in appendix C. In figure 6, we show the performance of MPC based on E-SINDy models for different training data length and noise=0.01. In figure 6*a*, we show the sensitivity of the mean MPC cost to training data length. We run 1000 noise realizations and average the mean MPC cost of all runs. Figure 6*b* shows trajectories of the controlled Lorenz system for models trained with E-SINDy and SINDy, using 50 and 150 time-step data points. E-SINDy significantly improves the MPC performance in the low data limit compared with SINDy.

## 5. Discussion

This work has developed and demonstrated a robust variant of the SINDy algorithm based on ensembling. The proposed E-SINDy algorithm significantly improves the robustness and accuracy of SINDy for model discovery, reducing the data requirements and increasing noise tolerance. E-SINDy exploits foundational statistical methods, such as bootstrap aggregating, to identify ensembles of ODEs and PDEs that govern the dynamics from noisy data. From this ensemble of models, aggregate model statistics are used to generate inclusion probabilities of candidate functions, which promotes interpretability in model selection and provides probabilistic forecasts. We show that ensembling may be used to improve several standard SINDy variants, including the integral formulation for PDEs. Combining ensembling with the integral formulation of SINDy enables the identification of PDE models from data with more than twice as much measurement noise as has been previously reported. These results are promising for the discovery of governing equations for complex systems in neuroscience, power grids, epidemiology, finance or ecology, where governing equations have remained elusive. Importantly, the computational effort to generate probabilistic models using E-SINDy is low. E-SINDy produces accurate probabilistic models in seconds, compared with existing Bayesian inference methods that take several hours. Library bagging has the additional advantage of making the least-squares computation more efficient by sampling only small subsets of the library. E-SINDy has also been incorporated into the open-source PySINDy package [62,63] to promote reproducible research.

We also present results exploiting the ensemble statistics for active learning and control. Recent active exploration methods [89] and active learning of nonlinear system identification [90] suggest exploration techniques using trajectory planning to efficiently explore high uncertainty regions of the feature space. We use the uncertainty estimates of E-SINDy to explore high uncertainty regions that maximally inform the learning process. Active E-SINDy reduces the variance of the identified model coefficients, increases the success rate of identifying the correct model structure and reduces the model coefficient error compared with standard E-SINDy in the low data limit. Finally, we apply E-SINDy to improve nonlinear MPC. SINDy was recently used to generate models for real-time MPC of nonlinear systems. We show that E-SINDy can significantly reduce the training data required to identify a model, thus enabling control of nonlinear systems with constraints in the very low data limit. An exciting future extension of the computationally efficient probabilistic model discovery is to combine the active learning and MPC strategies based on E-SINDy. An important avenue of future work may also explore active sampling that is constrained by the physical limitations of a given simulation or experiment. Highly efficient exploration and identification of nonlinear models may also enable learning task-agnostic models that are fundamental components of model-based reinforcement learning.

## Figures and Tables

**Figure 7.**
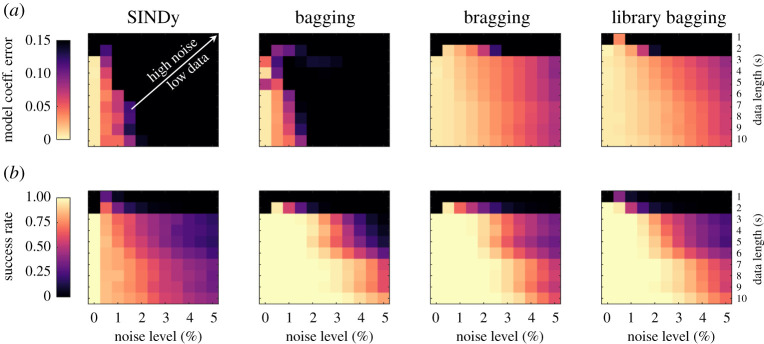
Noise level and data length sensitivity of different SINDy and ensemble SINDy models of the Lorenz system dynamics. (*a*) Model coefficient error and (*b*) success rate in identifying the correct model structure. Each noise level and data length case is run on 1000 realizations of noise and averaged for plotting. Ensembling improves the accuracy of SINDy with less data and more noise. (Online version in colour.)

**Figure 8.**
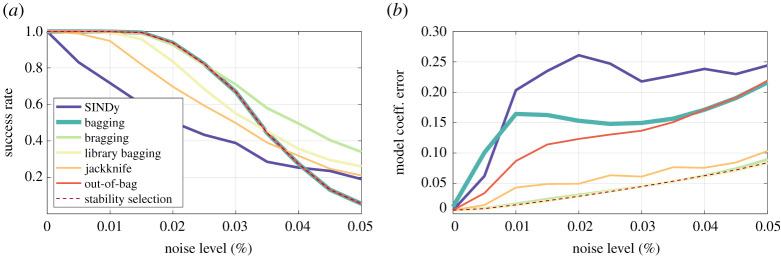
Detailed view on noise level sensitivity of SINDy and different ensemble SINDy models of the Lorenz system dynamics for 4s data length (using the same data as in figure 7 with each noise level run on 1000 realizations of noise and averaged for plotting). (*a*) Success rate in identifying the correct model structure and (*b*) model coefficient error. (Online version in colour.)

## Data Availability

This paper contains no experimental data. The E-SINDy method is implemented in the open-source software PySINDy, which is available at https://github.com/dynamicslab/pysindy. Additionally, the data and code to reproduce the results are available at https://github.com/urban-fasel/EnsembleSINDy.

## Footnotes

1 b(r)agging refers to robust bagging based on median, rather than mean, information.

## Appendix A. E-SINDy trends compared with standard SINDy

Here, we evaluate noise and data length sensitivity of E-SINDy compared with the standard SINDy implementation with no innovations for noise robustness. This case does not demonstrate the maximum noise robustness, but rather provides a simple and intuitive introduction to how E-SINDy compares with a baseline SINDy algorithm. E-SINDy can be used with most SINDy variants, and similar performance increases are found. For example, using integral SINDy or computing derivatives using the total variation derivative will dramatically improve the baseline robustness and therefore the E-SINDy robustness, as shown in §4b.

We define three commonly used metrics of success for identifying sparse nonlinear models: (1) the structure of the identified model, i.e. identifying the correct zero and non-zero terms in library; (2) the error of the identified model coefficients; and (3) the accuracy of predicted trajectories. The accuracy of a predicted trajectory is highly sensitive to small differences in the model coefficients, especially for chaotic systems. Therefore, we assess the structure of the identified model and the error of the identified coefficients quantitatively, and assess the accuracy of the forecast qualitatively. In figure 7, we compare the noise level and data length sensitivity of SINDy with bagging, bragging and library bagging E-SINDy, for data from the Lorenz system. We generate trajectories from the Lorenz system with system parameters shown in figure 1 and initial condition ${\text{u}}_{0}=[-8,\;7,\;27]$, for a range of data length. We then add Gaussian white noise with zero mean and variance $\sigma /||u|{|}_{\text{rms}}$, where $||u|{|}_{\text{rms}}$ is the root mean squared value of the trajectory data. We test each noise level and data length case with 1000 random realizations of noise, compute the success rate of identifying the correct model structure in Ξ and calculate the mean error of the identified model coefficients. The sparsity promoting hyperparameter is set to λ=0.2 for all methods. The number of models in the ensemble is set to q=100. The inclusion probability used to threshold model coefficients is set to tol=0.6 for b(r)agging and tol=0.4 for library bagging. We choose a smaller threshold for library bagging because we perform bagging after library bagging. Thus, the goal is not to aggressively threshold the identified coefficients, but only to reduce the initial library size to simplify the complexity of the subsequent bagging E-SINDy. We found that this strategy can improve the success rate and reduce the model error. Additionally, in the case of library bagging, we define the number of library terms in each sample to be l=0.6D.

The results for the different ensemble methods over noise level and data length are shown in figure 7. We see that ensembling clearly improves the accuracy and robustness of the model discovery, enabling model identification with less data and more noise than standard SINDy. As expected, bragging further robustifies bagging, and library bagging also improves accuracy and robustness. Additionally, library bagging has the advantage that we can consider larger libraries, discarding irrelevant terms in the library before applying b(r)agging E-SINDy. In the low data limit with little or no noise, we see that bagging does not significantly improve accuracy, and can even perform worse than standard SINDy. This is caused by outliers in the ensemble of identified models that decrease the accuracy of the aggregated model. We therefore suggest bragging, which robustifies bagging and improves the accuracy of the identified model by taking the median instead of the mean of the ensemble.

In terms of the accuracy of the E-SINDy forecast, we show library bagging for 2.5% noise and data length T=10s in the bottom of figure 1. Library bagging E-SINDy (coloured solid line) is able to forecast several chaotic lobe switches, compared with SINDy (grey dotted line), which deviates much earlier from the true dynamics (dashed line). We also show the 95% confidence intervals for different ensemble forecasting strategies, where multiple models are drawn from the bootstrap and used for prediction. Additionally, figure 1 shows the model coefficient distribution and inclusion probabilities of the identified library bagging E-SINDy model. The variance of the identified coefficients is comparably low, and the inclusion probabilities for the non-zero terms are clearly higher than for the zero terms.

Alternative resampling strategies such as jackknife [91] or using out-of-bag error estimates [92] may also improve the robustness of model discovery. In jackknife, we sequentially remove one observation from the data and identify an ensemble of models. This method may efficiently be used with library bagging, however, we found that bootstrapping outperforms jackknife, which is also reported in the literature [91]. We may also use out-of-bag error estimates to improve bagging. In bagging, each bootstrap sample leaves out approximately 37% of the data. This data can be used to give error estimates of the bagged predictor. We tested out-of-bag bagging E-SINDy, where we only aggregate the 10% most accurate models in terms of out-of-bag error. We found improved performance compared with bagging, however, we found lower accuracy and success rate compared with bragging. The more recent stability selection method [78] is another alternative to bagging and bragging. We perform the same sensitivity analysis to noise level and data length using the stability selection method that was recently used for PDE model discovery in [69]. Stability selection can be used to automatically determine the level of regularization in SINDy. The method randomly samples data subsets of size m/2 without replacement, identifies SINDy models for each subset, computes an importance measure and thresholds library terms, and solves a final linear least-squares regression on the thresholded library. The method in its original form performs slightly worse than b(r)agging E-SINDy. However, the success rate of stability selection SINDy can be improved, achieving comparable performance with bragging E-SINDy, by sampling subsets of size m with replacement instead of m/2 without replacement. In figure 8, we show the noise level sensitivity of the different ensemble SINDy methods including jackknife, out-of-bag bagging and stability selection of the Lorenz system dynamics for 4s data length.

## Appendix B. PDEs

Here, we describe the numerical methods and discretizations for creating the data used in the PDE model discovery results in §4b. The code to generate the data and reproduce the results can be found in our E-SINDy data and code repository https://github.com/urban-fasel/EnsembleSINDy. We use the data, numerical methods and spatial and temporal discretizations from [17,54].

The numerical solutions are obtained for $(x,t)\in x_{b}\times t_{b}$ and spatial- and temporal discretization n and m with periodic boundary conditions using Fourier-spectral differentiation and a Runge–Kutta-45 ODE solver (except for the inviscid Burgers where exact data are taken from a shock-forming solution described in [54]). The domain discretizations for the different examples are given in table 1.

**Table 1.** Spatial and temporal discretizations for different PDEs.

PDE	n	m	$x_{b}$	$t_{b}$
Inviscid Burgers	256	256	[−4000,4000]	[0, 4]
Korteweg de Vries	400	601	[−π,π]	[0, 0.006]
nonlinear Schroedinger	256	251	[−5,5]	[0,π]
Kuramoto–Sivashinsky	256	301	[0, 100]	[0, 150]
reaction–diffusion	256×256	201	[−10,10]×[−10,10]	[0, 4]

## Appendix C. MPC

In MPC, we compute a control sequence $u(x_{j})=\{u_{j+1},\ldots ,u_{j+m_{c}}\}$, given the current state estimate or measurement $x_{j}$, by a constrained optimization over a receding horizon $T_{c}=m_{c}\Delta t$, with Δt the time step of the model and $m_{c}$ the number of time steps. At each time step, we repeat the optimization, update the control sequence over the control horizon and apply the first control action $u_{j+1}$ to the system. The optimal control sequence $u(x_{j})$ is obtained by minimizing a cost function J over a prediction horizon $T_{p}=m_{p}\Delta t\geq T_{c}$. The cost function is

$$J=\sum_{k=0}^{m_{p}-1}||{\hat{\text{x}}}_{j+k}-{\text{r}}_{k}|{|}_{\text{Q}}^{2}+\sum_{k=1}^{m_{c}-1}(||{\text{u}}_{j+k}|{|}_{{\text{R}}_{u}}^{2}+||\Delta {\text{u}}_{j+k}|{|}_{{\text{R}}_{\Delta u}}^{2}),$$

subject to the discrete-time dynamics and constraints. The cost function J penalizes deviations of the predicted state $\hat{x}$ from the reference trajectory r, the control expenditure u and the rate of change of the control signal Δu. Each term is weighted by the matrices Q, ${\text{R}}_{u}$ and ${\text{R}}_{\Delta u}$, respectively.

Here, we use E-SINDy to identify a model of the forced Lorenz system dynamics and use MPC to stabilize one of the two unstable fixed points. The Lorenz dynamics are given by

C 1a $$\begin{array}{rl} \dot{x} & \;=\sigma (y-x)+u \end{array}$$

C 1b $$\begin{array}{rl} \dot{y} & \;=x(\rho -z)-y \end{array}$$

C 1c $$\begin{array}{rl} \mathrm{and}\; & \dot{z}=xy-\beta z \end{array}$$

with system parameters σ=10, β=8/3, ρ=28, and control input u affecting only the first state. The MPC objective is to stabilize one of the two unstable fixed points $\left(\pm \sqrt{72},\pm \sqrt{72},27\right)$. The weight matrices are $Q=I_{3}$, $R_{u}=R_{\Delta u}=0.001$, and the actuation input is limited to u∈[−50,50]. The control and prediction horizon is $m_{p}=m_{c}=5$ and the sparsity-promoting parameter in SINDYc is λ=0.2. We train the model with a Schroeder sweep and show the performance of the MPC for different training data length in figure 6. We show trajectories of the controlled Lorenz system for models trained with E-SINDy and SINDy and show that E-SINDy significantly improves the MPC performance in the low data limit compared with SINDy.

## References

[1] Kevrekidis IG, Gear CW, Hyman JM, Kevrekidis PG, Runborg O, Theodoropoulos C. 2003 Equation-free, coarse-grained multiscale computation: enabling mocroscopic simulators to perform system-level analysis. Commun. Math. Sci. **1**, 715-762. (10.4310/CMS.2003.v1.n4.a5)

[2] Majda AJ, Franzke C, Crommelin D. 2009 Normal forms for reduced stochastic climate models. Proc. Natl Acad. Sci. USA **106**, 3649-3653. (10.1073/pnas.0900173106) 19228943 PMC2645348

[3] Yair O, Talmon R, Coifman RR, Kevrekidis IG. 2017 Reconstruction of normal forms by learning informed observation geometries from data. Proc. Natl Acad. Sci. USA **114**, E7865-E7874. (10.1073/pnas.1620045114) 28831006 PMC5617245

[4] Kalia M, Brunton SL, Meijer HG, Brune C, Kutz JN. 2021 Learning normal form autoencoders for data-driven discovery of universal, parameter-dependent governing equations. Preprint. (https://arxiv.org/abs/2106.05102)

[5] Giannakis D, Majda AJ. 2012 Nonlinear Laplacian spectral analysis for time series with intermittency and low-frequency variability. Proc. Natl Acad. Sci. USA **109**, 2222-2227. (10.1073/pnas.1118984109) 22308430 PMC3289315

[6] Mezić I. 2005 Spectral properties of dynamical systems, model reduction and decompositions. Nonlinear Dyn. **41**, 309-325. (10.1007/s11071-005-2824-x)

[7] Mezic I. 2013 Analysis of fluid flows via spectral properties of the Koopman operator. Annu. Rev. Fluid Mech. **45**, 357-378. (10.1146/fluid.2013.45.issue-1)

[8] Schmid PJ. 2010 Dynamic mode decomposition of numerical and experimental data. J. Fluid Mech. **656**, 5-28. (10.1017/S0022112010001217)

[9] Rowley CW, Mezić I, Bagheri S, Schlatter P, Henningson DS. 2009 Spectral analysis of nonlinear flows. J. Fluid Mech. **645**, 115-127. (10.1017/S0022112009992059)

[10] Kutz JN, Brunton SL, Brunton BW, Proctor JL. 2016 Dynamic mode decomposition: data-driven modeling of complex systems. Philadelphia, PA: SIAM.

[11] Bongard J, Lipson H. 2007 Automated reverse engineering of nonlinear dynamical systems. Proc. Natl Acad. Sci. USA **104**, 9943-9948. (10.1073/pnas.0609476104) 17553966 PMC1891254

[12] Schmidt M, Lipson H. 2009 Distilling free-form natural laws from experimental data. Science **324**, 81-85. (10.1126/science.1165893) 19342586

[13] Schmidt MD, Vallabhajosyula RR, Jenkins JW, Hood JE, Soni AS, Wikswo JP, Lipson H. 2011 Automated refinement and inference of analytical models for metabolic networks. Phys. Biol. **8**, 055011. (10.1088/1478-3975/8/5/055011) 21832805 PMC4109817

[14] Daniels BC, Nemenman I. 2015 Automated adaptive inference of phenomenological dynamical models. Nat. Commun. **6**, 8133. (10.1038/ncomms9133) 26293508 PMC4560822

[15] Daniels BC, Nemenman I. 2015 Efficient inference of parsimonious phenomenological models of cellular dynamics using S-systems and alternating regression. PLoS ONE **10**, e0119821. (10.1371/journal.pone.0119821) 25806510 PMC4373916

[16] Brunton SL, Proctor JL, Kutz JN. 2016 Discovering governing equations from data by sparse identification of nonlinear dynamical systems. Proc. Natl Acad. Sci. USA **113**, 3932-3937. (10.1073/pnas.1517384113) 27035946 PMC4839439

[17] Rudy SH, Brunton SL, Proctor JL, Kutz JN. 2017 Data-driven discovery of partial differential equations. Sci. Adv. **3**, e1602614. (10.1126/sciadv.1602614) 28508044 PMC5406137

[18] Raissi M, Perdikaris P, Karniadakis GE. 2017 Machine learning of linear differential equations using Gaussian processes. J. Comput. Phys. **348**, 683-693. (10.1016/j.jcp.2017.07.050)

[19] Gottwald GA, Reich S. 2021 Supervised learning from noisy observations: combining machine-learning techniques with data assimilation. Physica D **423**, 132911. (10.1016/j.physd.2021.132911) 34717332

[20] Gottwald GA, Reich S. 2021 Combining machine learning and data assimilation to forecast dynamical systems from noisy partial observations. Chaos **31**, 101103. (10.1063/5.0066080) 34717332

[21] Raissi M, Perdikaris P, Karniadakis GE. 2019 Physics-informed neural networks: a deep learning framework for solving forward and inverse problems involving nonlinear partial differential equations. J. Comput. Phys. **378**, 686-707. (10.1016/j.jcp.2018.10.045)

[22] Chen RT, Rubanova Y, Bettencourt J, Duvenaud D. 2018 Neural ordinary differential equations. Preprint. (https://arxiv.org/abs/1806.07366)

[23] Champion K, Lusch B, Kutz JN, Brunton SL. 2019 Data-driven discovery of coordinates and governing equations. Proc. Natl Acad. Sci. USA **116**, 22 445-22 451. (10.1073/pnas.1906995116) PMC6842598 31636218

[24] Li Z, Kovachki N, Azizzadenesheli K, Liu B, Bhattacharya K, Stuart A, Anandkumar A. 2020 Fourier neural operator for parametric partial differential equations. Preprint. (https://arxiv.org/abs/2010.08895)

[25] Rackauckas C, Ma Y, Martensen J, Warner C, Zubov K, Supekar R, Skinner D, Ramadhan A. 2020 Universal differential equations for scientific machine learning. Preprint. (https://arxiv.org/abs/2001.04385)

[26] Lu L, Jin P, Pang G, Zhang Z, Karniadakis GE. 2021 Learning nonlinear operators via DeepONet based on the universal approximation theorem of operators. Nat. Mach. Intell. **3**, 218-229. (10.1038/s42256-021-00302-5)

[27] Long Z, Lu Y, Ma X, Dong B. 2018 PDE-net: learning PDEs from Data. In *Int. Conf. on Machine Learning*, pp. 3208–3216. PMLR.

[28] Breiman L. 1996 Bagging predictors. Mach. Learn. **24**, 123-140. (10.1007/BF00058655)

[29] Schaeffer H. 2017 Learning partial differential equations via data discovery and sparse optimization. *Proc. R. Soc. A* 473, 20160446. (10.1098/rspa.2016.0446) PMC5312119 28265183

[30] Loiseau JC, Brunton SL. 2018 Constrained sparse Galerkin regression. J. Fluid. Mech. **838**, 42-67. (10.1017/jfm.2017.823)

[31] Loiseau JC, Noack BR, Brunton SL. 2018 Sparse reduced-order modeling: sensor-based dynamics to full-state estimation. J. Fluid Mech. **844**, 459-490. (10.1017/jfm.2018.147)

[32] Loiseau JC. 2020 Data-driven modeling of the chaotic thermal convection in an annular thermosyphon. Theor. Comput. Fluid Dyn. **34**, 339-365. (10.1007/s00162-020-00536-w)

[33] Guan Y, Brunton SL, Novosselov I. 2021 Sparse nonlinear models of chaotic electroconvection. R. Soc. Open Sci. **8**, 202367. (10.1098/rsos.202367) 34430040 PMC8355675

[34] Deng N, Noack BR, Morzyński M, Pastur LR. 2021 Galerkin force model for transient and post-transient dynamics of the fluidic pinball. J. Fluid Mech. **918**, 1-37. (10.1017/jfm.2021.299)

[35] Callaham JL, Rigas G, Loiseau JC, Brunton SL. 2021 An empirical mean-field model of symmetry-breaking in a turbulent wake. Preprint. (https://arxiv.org/abs/2105.13990) 10.1098/rspa.2021.0092 PMC8299553 35153564

[36] Dam M, Brøns M, Juul Rasmussen J, Naulin V, Hesthaven JS. 2017 Sparse identification of a predator-prey system from simulation data of a convection model. Phys. Plasmas **24**, 022310. (10.1063/1.4977057)

[37] Alves EP, Fiuza F. 2020 Data-driven discovery of reduced plasma physics models from fully-kinetic simulations. Preprint. (https://arxiv.org/abs/2011.01927)

[38] Kaptanoglu AA, Morgan KD, Hansen CJ, Brunton SL. 2021 Physics-constrained, low-dimensional models for MHD: first-principles and data-driven approaches. Phys. Rev. E **104**, 015206. (10.1103/PhysRevE.104.015206) 34412353

[39] Beetham S, Capecelatro J. 2020 Formulating turbulence closures using sparse regression with embedded form invariance. Phys. Rev. Fluids **5**, 084611. (10.1103/PhysRevFluids.5.084611)

[40] Beetham S, Fox RO, Capecelatro J. 2021 Sparse identification of multiphase turbulence closures for coupled fluid–particle flows. J. Fluid Mech. **914**, 1-23. (10.1017/jfm.2021.53)

[41] Schmelzer M, Dwight RP, Cinnella P. 2020 Discovery of algebraic Reynolds-stress models using sparse symbolic regression. Flow Turbul. Combust. **104**, 579-603. (10.1007/s10494-019-00089-x)

[42] Zanna L, Bolton T. 2020 Data-driven equation discovery of ocean mesoscale closures. Geophys. Res. Lett. **47**, e2020GL088376. (10.1029/2020GL088376)

[43] Sorokina M, Sygletos S, Turitsyn S. 2016 Sparse identification for nonlinear optical communication systems: SINO method. Opt. Express **24**, 30 433-30 443. (10.1364/OE.24.030433) 28059391

[44] Boninsegna L, Nüske F, Clementi C. 2018 Sparse learning of stochastic dynamical equations. J. Chem. Phys. **148**, 241723. (10.1063/1.5018409) 29960307

[45] Thaler S, Paehler L, Adams NA. 2019 Sparse identification of truncation errors. J. Comput. Phys. **397**, 108851. (10.1016/j.jcp.2019.07.049)

[46] Kaiser E, Kutz JN, Brunton SL. 2018 Sparse identification of nonlinear dynamics for model predictive control in the low-data limit. Proc. R. Soc. A. **474**, 20180335. (10.1098/rspa.2018.0335) 30839858 PMC6283900

[47] Mangan NM, Brunton SL, Proctor JL, Kutz JN. 2016 Inferring biological networks by sparse identification of nonlinear dynamics. IEEE Trans. Mol. Biol. Multi-Scale Commun. **2**, 52-63. (10.1109/TMBMC.2016.2633265)

[48] Kaheman K, Kutz JN, Brunton SL. 2020 SINDy-PI: a robust algorithm for parallel implicit sparse identification of nonlinear dynamics. Proc. R. Soc. A **476**, 20200279. (10.1098/rspa.2020.0279) 33214760 PMC7655768

[49] Kaptanoglu AA, Callaham JL, Hansen CJ, Aravkin A, Brunton SL. 2021 Promoting global stability in data-driven models of quadratic nonlinear dynamics. Phys. Rev. Fluids **6**, 094401. (10.1103/PhysRevFluids.6.094401)

[50] Schaeffer H, McCalla SG. 2017 Sparse model selection via integral terms. Phys. Rev. E **96**, 023302. (10.1103/PhysRevE.96.023302) 28950639

[51] Reinbold PA, Gurevich DR, Grigoriev RO. 2020 Using noisy or incomplete data to discover models of spatiotemporal dynamics. Phys. Rev. E **101**, 010203. (10.1103/PhysRevE.101.010203) 32069592

[52] Reinbold PA, Kageorge LM, Schatz MF, Grigoriev RO. 2021 Robust learning from noisy, incomplete, high-dimensional experimental data via physically constrained symbolic regression. Nat. Commun. **12**, 1-8. (10.1038/s41467-021-23479-0) 34050155 PMC8163752

[53] Messenger DA, Bortz DM. 2021 Weak SINDy: Galerkin-based data-driven model selection. Multiscale Model. Simul. **19**, 1474-1497. (10.1137/20M1343166) PMC10795802 38239761

[54] Messenger DA, Bortz DM. 2021 Weak SINDy for partial differential equations. J. Comput. Phys. **443**, 110525. (10.1016/j.jcp.2021.110525) 34744183 PMC8570254

[55] Callaham JL, Loiseau JC, Rigas G, Brunton SL. 2021 Nonlinear stochastic modelling with Langevin regression. Proc. R. Soc. A **477**, 20210092. (10.1098/rspa.2021.0092) 35153564 PMC8299553

[56] Gelß P, Klus S, Eisert J, Schütte C. 2019 Multidimensional approximation of nonlinear dynamical systems. J. Comput. Nonlinear Dyn. **14**, 061006. (10.1115/1.4043148)

[57] Galioto N, Gorodetsky AA. 2020 Bayesian system id: optimal management of parameter, model, and measurement uncertainty. Nonlinear Dyn. **102**, 241-267. (10.1007/s11071-020-05925-8)

[58] Niven RK, Mohammad-Djafari A, Cordier L, Abel M, Quade M. 2020 Bayesian identification of dynamical systems. In *Multidisciplinary digital publishing institute Proc.*, *39th International Workshop on Bayesian Inference and Maximum Entropy Methods in Science and Engineering**,* *Garching, Germany,* *30 June–5 July*, vol. 33, p. 33.

[59] Yang Y, Aziz Bhouri M, Perdikaris P. 2020 Bayesian differential programming for robust systems identification under uncertainty. Proc. R. Soc. A **476**, 20200290. (10.1098/rspa.2020.0290) 33362409 PMC7735302

[60] Hirsh SM, Barajas-Solano DA, Kutz JN. 2021 Sparsifying priors for bayesian uncertainty quantification in model discovery. Preprint. (https://arxiv.org/abs/2107.02107) 10.1098/rsos.211823 PMC8864363 35223066

[61] Delahunt CB, Kutz JN. 2021 A toolkit for data-driven discovery of governing equations in high-noise regimes. Preprint. (https://arxiv.org/abs/2111.04870)

[62] de Silva BM, Champion K, Quade M, Loiseau JC, Kutz JN, Brunton SL. 2020 PySINDy: a python package for the sparse identification of nonlinear dynamics from data. J. Open Source Softw. **5**, 2104. (10.21105/joss)

[63] Kaptanoglu AA *et al.* 2021 PySINDy: a comprehensive Python package for robust sparse system identification. Preprint (https://arxiv.org/abs/2111.08481). 10.1103/PhysRevE.104.015206 34412353

[64] Gurevich DR, Reinbold PA, Grigoriev RO. 2019 Robust and optimal sparse regression for nonlinear PDE models. Chaos **29**, 103113. (10.1063/1.5120861) 31675826

[65] Gurevich DR, Reinbold PA, Grigoriev RO. 2021 Learning fluid physics from highly turbulent data using sparse physics-informed discovery of empirical relations (SPIDER). Preprint (https://arxiv.org/abs/2105.00048).

[66] Sashidhar D, Kutz JN. 2021 Bagging, optimized dynamic mode decomposition (BOP-DMD) for robust, stable forecasting with spatial and temporal uncertainty-quantification. Preprint (https://arxiv.org/abs/2107.10878).

[67] Sachdeva PS, Livezey JA, Tritt AJ, Bouchard KE. 2019 Pyuoi: the union of intersections framework in python. J. Open Source Softw. **4**, 1799. (10.21105/joss)

[68] Nardini JT, Lagergren JH, Hawkins-Daarud A, Curtin L, Morris B, Rutter EM, Swanson KR, Flores KB. 2020 Learning equations from biological data with limited time samples. Bull. Math. Biol. **82**, 1-33. (10.1007/s11538-020-00794-z) PMC8409251 32909137

[69] Maddu S, Cheeseman BL, Sbalzarini IF, Müller CL. 2019 Stability selection enables robust learning of partial differential equations from limited noisy data. Preprint (https://arxiv.org/abs/1907.07810).

[70] Towne A, Schmidt OT, Colonius T. 2018 Spectral proper orthogonal decomposition and its relationship to dynamic mode decomposition and resolvent analysis. J. Fluid Mech. **847**, 821-867. (10.1017/jfm.2018.283)

[71] Champion K, Zheng P, Aravkin AY, Brunton SL, Kutz JN. 2020 A unified sparse optimization framework to learn parsimonious physics-informed models from data. IEEE Access **8**, 169 259-169 271. (10.1109/Access.6287639)

[72] Carderera A, Pokutta S, Schütte C, Weiser M. 2021 CINDy: conditional gradient-based Identification of non-linear dynamics–noise-robust recovery. Preprint (https://arxiv.org/abs/2101.02630).

[73] Bühlmann PL. 2003 Bagging, subagging and bragging for improving some prediction algorithms. In *Research report/seminar für statistik, eidgenössische technische hochschule (ETH)*, vol. 113. Zürich: Seminar für Statistik, Eidgenössische Technische Hochschule (ETH).

[74] Bühlmann P. 2012 Bagging, boosting and ensemble methods. In *Handbook of computational statistics*, pp. 985–1022. Berlin, Germany: Springer.

[75] Schapire RE. 1990 The strength of weak learnability. Mach. Learn. **5**, 197-227. (10.1007/BF00116037)

[76] Freund Y. 1995 Boosting a weak learning algorithm by majority. Inf. Comput. **121**, 256-285. (10.1006/inco.1995.1136)

[77] Hastie T, Tibshirani R, Friedman J. 2001 The elements of statistical learning. Springer Series in Statistics. Berlin, Germany: Springer.

[78] Meinshausen N, Bühlmann P. 2010 Stability selection. J. R. Stat. Soc. Series B Stat. Methodol. **72**, 417-473. (10.1111/rssb.2010.72.issue-4)

[79] Hewitt CG. 1921 The conservation of the wild life of Canada. New York, NY: C. Scribner.

[80] Krogh A, Vedelsby J. 1995 Neural network ensembles, cross validation, and active learning. Adv. Neural Inf. Process. Syst. **7**, 231-238.

[81] Zhu X, Zhang P, Lin X, Shi Y. 2010 Active learning from stream data using optimal weight classifier ensemble. IEEE Trans. Syst. Man Cybern. B **40**, 1607-1621. (10.1109/TSMCB.2010.2042445) 20363683

[82] Settles B. 2009 Active learning literature survey. University of Wisconsin-Madison, Department of Computer Sciences.

[83] Settles B. 2011 From theories to queries: active learning in practice. In *Active learning and experimental design workshop in conjunction with AISTATS 2010,* *JMLR Workshop and Conf*. *Proc.*, pp. 1–18.

[84] Garcia CE, Prett DM, Morari M. 1989 Model predictive control: theory and practice—a survey. Automatica **25**, 335-348. (10.1016/0005-1098(89)90002-2)

[85] Morari M, Lee JH. 1999 Model predictive control: past, present and future. Comput. Chem. Eng. **23**, 667-682. (10.1016/S0098-1354(98)00301-9)

[86] Mayne DQ. 2014 Model predictive control: recent developments and future promise. Automatica **50**, 2967-2986. (10.1016/j.automatica.2014.10.128)

[87] Peng H, Wu J, Inoussa G, Deng Q, Nakano K. 2009 Nonlinear system modeling and predictive control using the RBF nets-based quasi-linear ARX model. Control Eng. Pract. **17**, 59-66. (10.1016/j.conengprac.2008.05.005)

[88] Zhang T, Kahn G, Levine S, Abbeel P. 2016 Learning deep control policies for autonomous aerial vehicles with MPC-guided policy search. In *IEEE Int. Conf. Rob. Autom*, *Stockholm,* *Sweden, May 16–21,* pp. 528–535.

[89] Shyam P, Jaśkowski W, Gomez F. 2019 Model-based active exploration. In *Int. Conf. on Machine Learning*, pp. 5779–5788. PMLR.

[90] Mania H, Jordan MI, Recht B. 2020 Active learning for nonlinear system identification with guarantees. Preprint. (https://arxiv.org/abs/2006.10277)

[91] Efron B. 1982 The jackknife, the bootstrap and other resampling plans. Philadelphia, PA: SIAM.

[92] Breiman L. 1996 Out-of-bag estimation. University of California, Statistics Department.

